# Development of Porous Epoxy Micro-Beads Using Ammonium Bicarbonate through a Single Epoxy Droplet in Corn Oil

**DOI:** 10.3390/ma14092282

**Published:** 2021-04-28

**Authors:** Anusha Leemsuthep, Zunaida Zakaria, Varaporn Tanrattanakul, Suganti Ramarad, Mathialagan Muniyadi, Tomasz Jaruga, Yamuna Munusamy, Izabela Wnuk, Paweł Pietrusiewicz

**Affiliations:** 1 Faculty of Chemical Engineering Technology, Universiti Malaysia Perlis (UniMAP), Arau 02600, Malaysia; anushaleem@gmail.com; 2 Geopolymer & Green Technology, Centre of Excellence (CEGeoGTech), Universiti Malaysia Perlis (UniMAP), Arau 02600, Malaysia; 3 Research and Development Office, Sino-Thai International Rubber College, Prince of Songkla University, Songkhla 90110, Thailand; varaporn.t@psu.ac.th; 4 School of Engineering and Physical Sciences, Heriot-Watt University Malaysia, Putrajaya 62200, Malaysia; R.Suganti@hw.ac.uk; 5 Department of Petrochemical Engineering, Faculty of Engineering and Green Technology, Universiti Tunku Abdul Rahman (Perak Campus), Jalan Universiti, Bandar Barat, Kampar 31900, Malaysia; mathialagan@utar.edu.my (M.M.); yamunam@utar.edu.my (Y.M.); 6 Faculty of Mechanical Engineering and Computer Science, Częstochowa University of Technology, 42201 Częstochowa, Poland; jaruga@ipp.pcz.pl; 7 Department of Physics, Częstochowa University of Technology, 42201 Częstochowa, Poland; wnuk.izabela@wip.pcz.pl (I.W.); pawel.pietrusiewicz@pcz.pl (P.P.)

**Keywords:** blowing agent, decomposition gas, emulsion, stoichiometry, thermal stability

## Abstract

This paper explored the effects of ammonium bicarbonate and different ratios of epoxy to polyamide on the formation of porous epoxy micro-beads through a single epoxy droplet. A single drop of a mixture, consisting of epoxy, polyamide, and ammonium bicarbonate, was dropped into heated corn oil at a temperature of 100 °C. An epoxy droplet was formed due to the immiscibility of the epoxy mixture and corn oil. The ammonium bicarbonate within this droplet underwent a decomposition reaction, while the epoxy and polyamide underwent a curing reaction, to form porous epoxy micro-beads. The result showed that the higher ammonium bicarbonate content in the porous, epoxy micro-beads increased the decomposition rate up to 11.52 × 10^−3^ cm^3^/s. In addition, a higher total volume of gas was generated when a higher ammonium bicarbonate content was decomposed. This led to the formation of porous epoxy micro-beads with a smaller particle size, lower specific gravity, and better thermal stability. At an epoxy to polyamide ratio of 10:6, many smaller micro-beads, with particle sizes ranging from 201 to 400 μm, were obtained at an ammonium bicarbonate content of 10 phr. Moreover, the porous epoxy micro-beads with open pores were shown to have a low specific gravity of about 0.93 and high thermal stability at a high ammonium bicarbonate content. Based on the findings, it was concluded that porous epoxy micro-beads were successfully produced using a single epoxy droplet in heated corn oil, where their shape and particle size depended on the content of ammonium bicarbonate and the ratio of epoxy to polyamide used.

## 1. Introduction

Porous micro-beads have been extensively commercialized in biomedical and structural applications due to their outstanding properties, such as low density, high surface area, high porosity, and economic value compared to solid micro-beads [1,2,3]. Their porous structure was composed of open pores or close pores. Kim and Lee [2] stated that a porous structure with a high storage capacity is an added feature for the separation of gas used in the membrane. In another research study by Li et al. [4], the porous micro-beads were used as adsorbents for the removal of dyes from aqueous solution. They claimed that this type of micro-beads increased the efficiency for absorption in solution. Various materials, such as glass, ceramic, metals, and polymers, have been used to produce porous micro-beads. The properties of the final product depend on the type of material used to produce the porous micro-beads [5]. Among the porous microbead materials, polymers dominate in many applications, including structural, automotive, electronics, and biomedical applications [6,7,8], as polymer-based micro-beads are inexpensive, and offer excellent thermal expansion and ease of fabrication [9,10]. Currently, porous epoxy micro-beads are being continuously explored by researchers due to their excellent mechanical and thermal properties, and superior chemical resistance due to the three-dimensional crosslinks formed by the reaction between epoxy and polyamide [11].

In numerous studies, the emulsion technique was used to produce porous micro-beads as it is simple and economical [12]. An emulsion is a system consisting of two or more immiscible liquid phases, wherein one liquid is dispersed in another liquid phase [13]. Droplets are formed when the latter surrounds the dispersed liquid. The most common emulsions provided by the single emulsion technique are water-in-oil (W/O) and oil-in-water (O/W) emulsions [14]. The formation of droplets plays an important role in emulsions and can affect the properties and morphology of the final product. Hence, a greater understanding of the parameters or properties for producing droplets is important.

In a previous study, Leemsuthep et al. [15] fabricated micro-porous epoxy using sodium bicarbonate using the single emulsion technique. Sodium bicarbonate was also used to produce calcium pectinate [16], carbon [17], hydrogel [18], and chitosan [19] micro-porous beads. In another work by Nayan et al. [11], the emulsion method was used to produce an epoxy micro-porous Porofor toluenesulfonylhydrazide 75 (Porofor TSH) blowing agent, which acted as a droplet expander or blower. Sodium bicarbonate and porofor TSH, as blowing agents, require a high temperature (140–160 °C) for decomposition. Similarly, a high temperature is required when blowing agents, such as azodicarbonamide, are used for the formation of micro-beads. Due to this aspect, a high temperature is required for the emulsion process, thereby, increasing the process cost. In addition, sodium bicarbonate produces a low percentage of gas and sodium carbonate as by-products. Blowing agents, such as azodicarbonamide, produce hydrazodicarbonamide and urazole as by-products [20], sodium carbonate produces sodium oxide as a by-product [21], and lithium carbonate produces lithium oxide as a by-product [22]. Additional chemicals or processes are required to eliminate the by-products. To overcome these drawbacks, ammonium bicarbonate was selected as the blowing agent in this study due to its decomposition at a low temperature and the absence of by-products. However, due to mechanical agitation by the stirrer introduced in a previous study by Leemsuthep et al. [15] and Nayan et al. [11], the clump of micro-porous was produced.

In this study, a single epoxy droplet was dropped into the heated corn oil to produce porous epoxy micro-beads. In order to understand the foaming mechanism, the fundamental study of the single droplet without mechanical agitation was investigated. In addition to the importance of a single epoxy droplet used, we highlight the effect of ammonium bicarbonate content and an epoxy polyamide ratio on the total micro-beads, microstructure, and particle size distribution of micro-beads produced. On the other hand, up to the present moment, we have not found any investigation that has studied production of micro-beads using a single epoxy droplet without mechanical agitation.

## 2. Experimental Section

### 2.1. Materials

Epoxy resin (grade DER 331) and polyamide (grade A062) were provided by Euro Chemo Pharma Sdn. Bhd (Pulau Pinang, Malaysia). Epoxy resin DER 331 contains epoxide at an equivalent weight of 182–192 with a density of 1.16 g/cm^3^ and viscosity of 11–14 Pas at 25 °C. Polyamide A062 has an equivalent weight per H active of 110, with a density of 0.96 g/cm^3^ and viscosity of 35–45 Pas at 25 °C. White crystalline ammonium bicarbonate powder, with a density of 1.58 g/cm^3^, was obtained from HmbG Chemicals (Hamburg, Germany) for use as a blowing agent. Corn oil, which was free of polyunsaturated fatty acids, was purchased from Yee Lee Corporation Sdn. Bhd (Ipoh, Perak, Malaysia).

### 2.2. Preparation of Porous Epoxy Micro-Bead (PEMB)

**Scheme 1:**
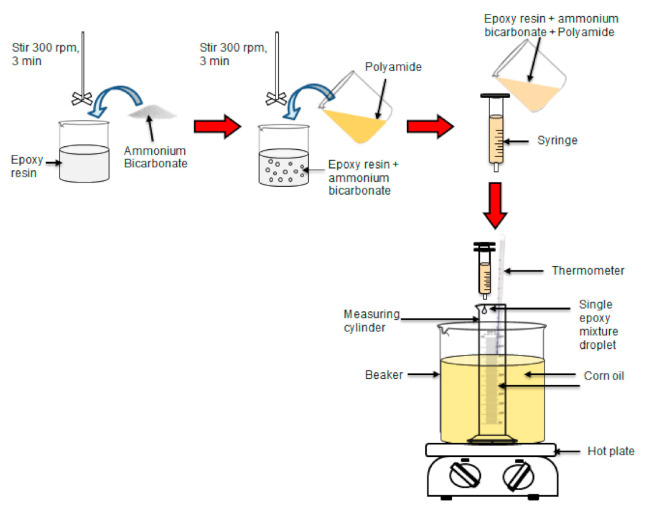
Summary of steps for the preparation of porous epoxy micro-beads.

Two different ratios of epoxy to polyamide (i.e., 10:6 and 10:10) were used in this study. The epoxy to polyamide ratio of 10:6 was calculated based on the stoichiometric ratio, whereas the ratio of 10:10 was similar to the stoichiometric ratio. The total weight of epoxy and polyamide was assigned as parts per hundred parts of resin (phr). The ammonium bicarbonate content varied between 0, 5, and 10 phr. First, epoxy and ammonium bicarbonate were mixed at a speed of 300 rpm for 3 min as shown in Scheme 1 using an Ika overhead stirrer (model RW20), supplied by A. R. Alatan Sdn. Bhd (Alor Setar, Kedah, Malaysia). Polyamide was added to the mixture and mixed at a speed of 300 rpm for another 3 min. Next, 0.1 mL of a single droplet of the epoxy mixture was dropped into 100 mL of heated corn oil in a measuring cylinder using a 5-mL/cc plastic syringe with a nozzle diameter of 2 mm. The heated corn oil was maintained at a temperature of 100 °C. The epoxy mixture droplet was left in the heated corn oil for 1 h for the foaming and curing reactions. The porous epoxy micro-beads (PEMBs) were collected and washed using detergent water at 60 °C. The ratio of detergent to water used was 1:20. The porous epoxy micro-beads were dried at 80 °C for 4 h in a Memmert conventional oven (model UF 30), supplied by A.R Alatan Sdn. Bhd [15]. A summary of the initial preparation steps is illustrated in Scheme 1.

### 2.3. Characterization and Testing

#### 2.3.1. Decomposition Rate and Total Volume of Gas Generated by Ammonium Bicarbonate

**Scheme 2:**
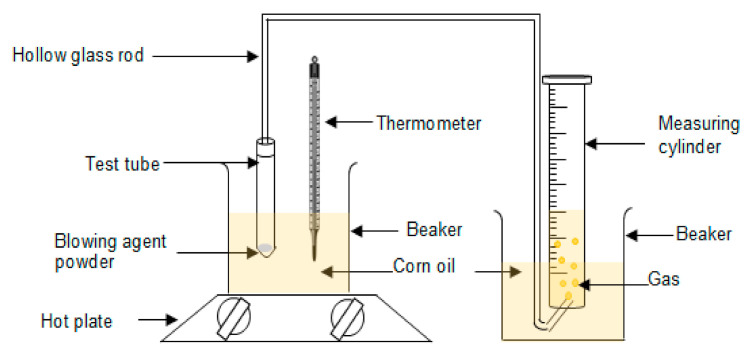
Setup of apparatus.

Ammonium bicarbonate with 5 and 10 phr were placed in the test tube and partially immersed in corn oil. The temperature of the corn oil was set at 100 °C. The amount of gas released through the hollow glass rod into the water in the measuring cylinder was recorded. The setup of the apparatus is shown in Scheme 2. The process was repeated for five cycles. The decomposition rate and total volume of gas generated by ammonium bicarbonate were determined by Equations (1) and (2).

(1) $$Decomposition\;rate=\frac{Volume\;of\;water\;displacement}{Time}$$

(2) $$Total\;volume\;of\;gas=\frac{Volume\;of\;water\;displacement}{Mass\;of\;ammonium\;bicarbonate}$$

#### 2.3.2. Morphology Analysis

The microstructure of the PEMBs was observed using a scanning electron microscope (SEM) (model JSM 6460 LA, from JEOL, Tokyo, Japan) to study the surface morphology, such as particle shape, size, and porous structure. A sputter-coating instrument was used to coat the samples with a layer of gold/palladium before the analysis to avoid electrostatic charging during the observation.

#### 2.3.3. Analysis of Particle Size

A Dino-lite digital microscope (model AM5216ZT Edge Series), supplied by Dpro Vision Sdn. Bhd. (Petaling Jaya, Selangor, Malaysia), equipped with a low-resolution digital camera using Dino Capture 2.0 software (Torrance, California) was used to capture the particle image from a single drop of epoxy mixture. The image was then analyzed using ImageJ software (Madison, WI, USA) to measure the particle size distribution of total micro-beads from a single epoxy droplet.

#### 2.3.4. Specific Gravity

A pycnometer bottle was used to determine the specific gravity (SG) of the PEMBs based on ASTM D854-14 [23]. The specific gravity (SG) of the PEMBs was calculated using Equation (3).

(3) $$SG=\frac{W_{2}-W_{1}}{\left(W_{2}-W_{1}\right)-\left(W_{3}-W_{4}\right)}$$

where *SG* is specific gravity, *W*_1_ is mass of pycnometer bottle, *W*_2_ is mass of pycnometer bottle filled with sample, *W*_3_ is mass of pycnometer bottle filled with sample and water, and *W*_4_ is mass of pycnometer bottle filled with water. The measurements were repeated five times.

#### 2.3.5. Thermal-Gravimetric Analysis

The thermal gravimetric analysis (TGA) was carried out using a Mettler Toledo TGA (Mettler Toledo, Switzerland). The purpose of the analysis was to establish the thermal properties of the samples along with their compositional properties. The heating rate of the samples was set at 20 °C/min, with temperatures ranging from room temperature to 600 °C under the flow of argon gas.

## 3. Results and Discussions

### 3.1. Decomposition Rate and Total Volume of Gas Generated by Ammonium Bicarbonate

**Figure 1:**
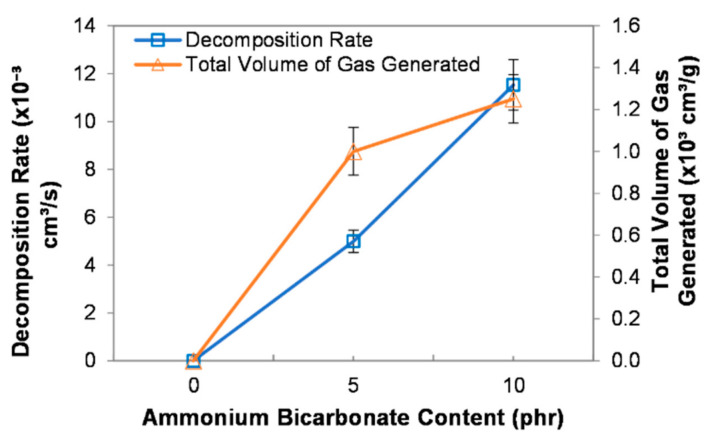
Decomposition rate and total volume of gas generated at different ammonium bicarbonate contents.

A single droplet of epoxy mixture, composed of epoxy, polyamide, and ammonium bicarbonate, was dropped into heated corn oil at a temperature of 100 °C. Once the droplet reached the desired temperature, it was blown and cured to form PEMBs. Ammonium bicarbonate played an essential role as a blowing agent, where the decomposition of ammonium bicarbonate generated gases, thereby, leading to the expansion of the droplet. Figure 1 shows the effect of ammonium bicarbonate content on the decomposition rate and the total volume of gas generated from the decomposition of ammonium bicarbonate. The decomposition rate of ammonium bicarbonate increased up to 11.52 × 10^−3^ cm^3^/s with increasing ammonium bicarbonate content from 0 to 10 phr. This could be implied that, at a higher ammonium bicarbonate content, the gas generated within one second will be higher. Therefore, a higher ammonium bicarbonate content will result in a higher decomposition rate.

The total volume of gas generated after the decomposition increased as the ammonium bicarbonate content increased. The decomposition of ammonium bicarbonate (NH_4_HCO_3_) produces ammonia (NH_3_), carbon dioxide (CO_2_), and water vapour (H_2_O). The decomposition of ammonium bicarbonate is shown in Equation (4) [24]. When a higher amount of ammonium bicarbonate was added, higher volumes of ammonia, carbon dioxide, and water vapour were generated [25]. Thus, the total volume of gas generated increased as the ammonium bicarbonate content increased.

NH_4_HCO_3_(s) + heat → NH_3_(g) + CO_2_(g) + H_2_O(g) (4)

### 3.2. Morphology Analysis

**Scheme 3:**
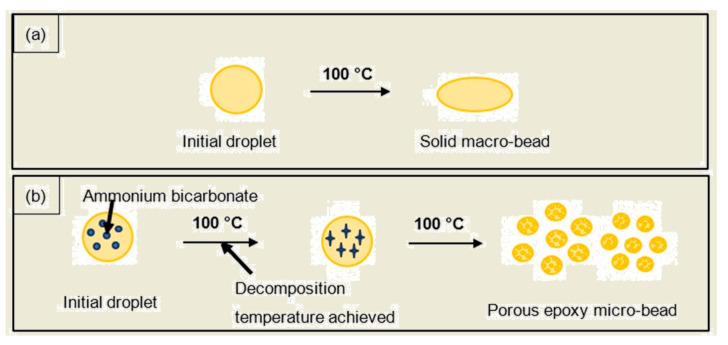
Schematic illustration of the formation of PEMBs from (**a**) epoxy-polyamide mixture, and (**b**) epoxy-polyamide-ammonium bicarbonate mixture.

The curing of the droplet started on the outer surface once it came into contact with the heated corn oil, and further curing was then propagated to the inner core [26]. Scheme 3 depicts the formation of the PEMBs, and illustrates how the initial droplet behaved in the heated corn oil. Scheme 3a shows that, when a single epoxy-polyamide droplet without ammonium bicarbonate was dropped into the heated corn oil, this droplet formed a sediment at the bottom of the measuring cylinder since no gases were generated in this droplet. Finally, the droplet was cured as a solid macro-bead. However, the addition of ammonium bicarbonate into the single epoxy mixture droplet caused this droplet to burst into many smaller droplets that were dispersed in the oil phase [15]. Once the decomposition temperature of ammonium bicarbonate was reached, the gases that were released as a result of the decomposition of ammonium bicarbonate created an internal pressure in the single epoxy mixture droplet. This internal pressure caused the single epoxy mixture droplet to burst into many smaller droplets, which were cured as PEMBs. The suggested mechanism is demonstrated in Scheme 3b.

**Figure 2:**
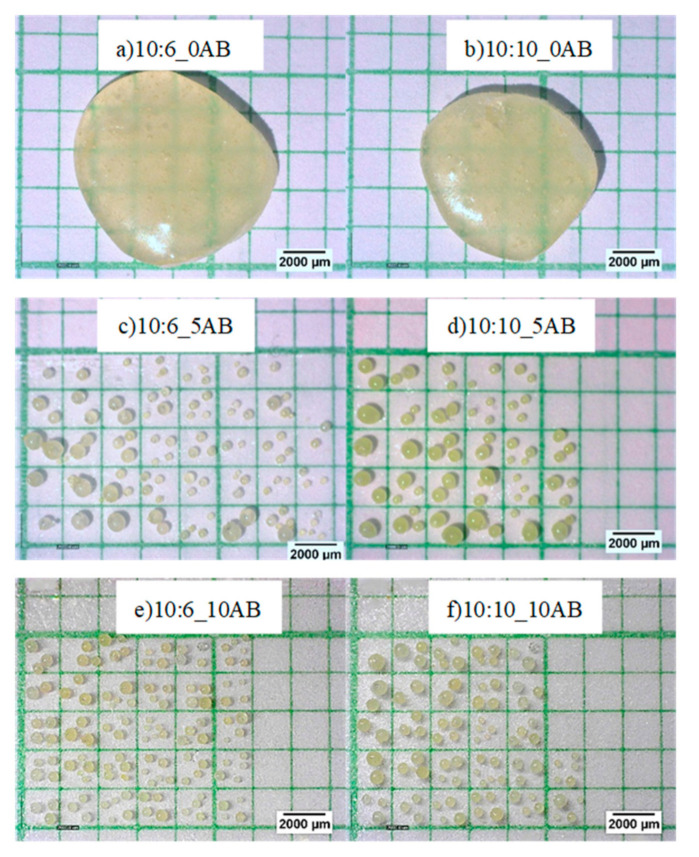
Morphology of PEMBs from a single droplet at different epoxy polyamide ratio and ammonium bicarbonate content (magnification of 20X): (**a**) 10:6_0AB, (**b**) 10:10_AB, (**c**)10:6_5AB, (**d**) 10:10_5AB, (**e**) 10:6_10AB, and (**f**) 10:10_10AB.

A single epoxy-polyamide droplet without ammonium bicarbonate was dropped into heated corn oil to produce a big particle. As no gases were produced in this droplet, it was unable to blow or expand, thus, producing a solid macro-bead after curing with a size of 8.86 mm for the sample with an epoxy to polyamide ratio of 10:6, and a size of 8.37 mm for the sample with an epoxy to polyamide ratio of 10:10. The addition of ammonium bicarbonate to the single droplet caused it to be blown into many smaller micro-beads, as can be seen in Figure 2, which shows the amount of PEMBs produced from the single droplet. The total of PEMBs from a single droplet increased by increasing the ammonium bicarbonate content from 5 phr (Figure 2c) to 10 phr (Figure 2e) for both epoxy polyamide ratios. As can be seen from the figure, the sample with an epoxy to polyamide ratio of 10:10 produced micro-beads with a larger particle size compared to the sample with an epoxy to polyamide ratio of 10:6. It was deemed that the excess of polyamide in a 10:10 ratio introduced free volume to the network structure. When ammonium bicarbonate decomposed, this network structure could hold the decomposed gas [27]. Therefore, a fewer number of PEMBs were formed from the sample with an epoxy to polyamide ratio of 10:10, and the particle size was also slightly larger than that of the PEMBs formed from the sample with an epoxy to polyamide ratio of 10:6.

**Figure 3:**
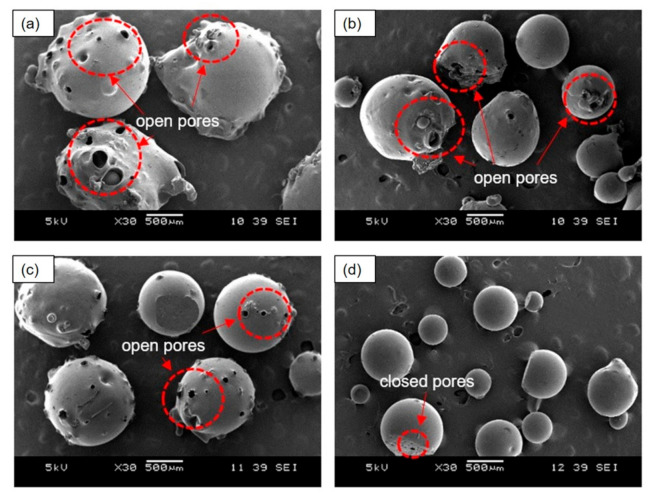
SEM images of PEMBs at different ammonium bicarbonate content and epoxy polyamide ratios at magnification of 30X: (**a**) 10:6_5AB, (**b**) 10:6_10AB, (**c**) 10:10_5AB, and (**d**) 10:10_10AB.

Figure 3 shows the morphology of the PEMBs prepared with different ammonium bicarbonate contents and epoxy to polyamide ratios. The results indicated that the samples with an epoxy to polyamide ratio of 10:6 with 5 phr of ammonium bicarbonate (10:6_5AB) and with an epoxy to polyamide ratio of 10:10 with 5 phr of ammonium bicarbonate (10:10_5AB) produced larger particles as compared to the samples with an epoxy to polyamide ratio of 10:6 with 10 phr of ammonium bicarbonate (10:6_10AB) and an epoxy to polyamide ratio of 10:10 with 10 phr of ammonium bicarbonate (10:10_10AB). This may have been due to the increase in the ammonium bicarbonate content from 0 to 10 phr, which caused the total volume of gases generated, following the decomposition of ammonium bicarbonate to be higher. This phenomenon induced a higher internal pressure in the single epoxy mixture droplet, which was then unable to withstand the gases that were generated, and, as a result, burst into many smaller open-pore micro-beads. For the 10:10_10AB sample, the micro-beads had closed pores, as shown in Figure 3d. It could be inferred that these droplets were able to withstand the internal pressure caused by the generated gases after the initial burst, thereby, accounting for the absence of pores on the surface of the micro-beads.

Furthermore, the ratio of epoxy to polyamide could have greatly affected the microstructure of the PEMBs. From Figure 3, the epoxy to polyamide ratio of 10:6 with 5 phr of ammonium bicarbonate produced irregular-shaped particles as compared to 10 phr of ammonium bicarbonate. Moreover, the epoxy to polyamide ratio of 10:10 for both ammonium bicarbonate contents produced particles that were more regular and spherical in shape. This was because the epoxy to polyamide ratio of 10:6 was dominant due to the formation of a three-dimensional network between the epoxide groups and amide groups as compared to the 10:10 ratio [28]. This characteristic resulted in short crosslinked chains and a low viscosity in the formulation with the ratio of 10:6 than with the ratio of 10:10. Due to this phenomenon, the single epoxy droplet was unable to withstand the decomposition gases and burst to form PEMBs with open pores.

### 3.3. Particle Size

**Figure 4:**
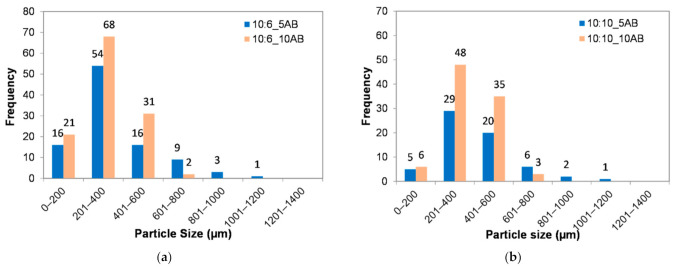
Histograms of particle size distributions of PEMBs at different ammonium bicarbonate contents and epoxy to polyamide ratios: (**a**) epoxy polyamide ratio of 10:6 and (**b**) epoxy polyamide ratio of 10:10.

Figure 4 shows the histograms of the PEMBs’ particle size distribution for the different ammonium bicarbonate contents and epoxy to polyamide ratios from a single epoxy droplet. Figure 4a shows that 10:6_5AB and 10:6_10AB sample produced microbeads with a uniform particle size distribution of between 201 to 400 μm for both a sample with means of 900.50 μm and 326.18 μm, respectively. Meanwhile, the 10:10_5AB and 10:10_10AB samples produced larger microbeads with a uniform particle size distribution between 201 to 400 μm as well with means of 1127.20 μm and 359.10 μm, respectively. When referring to Figure 4a,b, it can be seen that the dominant particle size of the microbeads shifted to the right site, when the content of ammonium bicarbonate was increased from 5 phr to 10 phr.

Furthermore, there were more PEMBs with a smaller particle size when the ammonium bicarbonate content was 10 phr than when it was 5 phr for both epoxy to polyamide ratios. This indicated that the higher ammonium bicarbonate content produced micro-beads with a smaller particle size. This may have been due to the high heat transfer caused by the presence of a large amount of ammonium bicarbonate. The probability of this heat being around the droplet was high due to the high volume of ammonium bicarbonate inside the droplet as compared to a low amount of ammonium bicarbonate, which tended to be encapsulated within the epoxy droplet. Therefore, 10 phr of ammonium bicarbonate tended to decompose and burst earlier, thereby, producing smaller particles as compared to the 6 phr of ammonium bicarbonate. Another reason could be that the 10 phr of ammonium bicarbonate produced a higher volume of gases. This induced a high internal pressure in the droplet, causing it to burst into many smaller micro-beads.

With regard to the effect of the epoxy to polyamide ratio, it was clear that the epoxy to polyamide ratio of 10:6 resulted in slightly smaller particles as compared to the epoxy to polyamide ratio of 10:10 with 5 phr of ammonium bicarbonate. Additionally, the epoxy to polyamide ratio of 10:10 produced a large, flexible, crosslinked, three-dimensional network because it had excessive polyamide in the epoxy-polyamide system. In other words, epoxy polyamide of a 10:10 ratio had more free volume in the structure [29]. This characteristic caused the decomposed gas to be entrapped in the structure, thereby, producing a larger particle size than the epoxy to polyamide ratio of 10:6.

### 3.4. Specific Gravity

**Figure 5:**
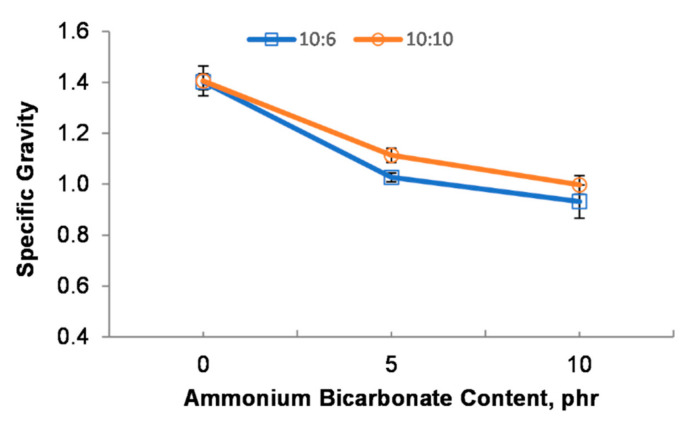
Specific gravity of PEMBs at different ammonium bicarbonate contents and epoxy polyamide ratios.

Figure 5 illustrates the relationship between the specific gravity and the ammonium bicarbonate loading with the epoxy to polyamide ratio. It was indicated that the specific gravity decreased as the ammonium bicarbonate content increased from 0 phr to 10 phr. The volume of gases generated by the decomposition of the blowing agent, and the microstructure and particle size of the micro-beads greatly influenced the specific gravity. As reported in Figure 5, the single epoxy-polyamide droplet (10:6_0AB and 10:10_0AB) without ammonium bicarbonate offered a higher specific gravity. The single epoxy-polyamide droplet was not blown and, thus, the droplet was cured as a solid macro-bead without any pores inside. This may explain the higher specific gravity that was obtained for the 10:6_0AB and 10:10_0AB samples. As expected, the addition of ammonium bicarbonate reduced the specific gravity. As mentioned in Section 3.1, the total volume of gases generated increased when the ammonium bicarbonate content was increased, leading to the production of many smaller porous particles and a reduction in the particle size. Subsequently, this reduced the specific gravity. This finding was also in agreement with References [30,31] in which the addition of a foaming agent in a polymer matrix can reduce the specific gravity of the polymer.

Additionally, different epoxy to polyamide ratios tended to influence the specific gravity as well. Based on Figure 5, the specific gravity of the 10:6_0AB sample was higher than that of the 10:10_0AB sample. The 10:6_0AB sample produced micro-beads with a larger particle size as compared to the 10:10_0AB sample. This could have caused the higher specific gravity of the 10:6_0AB sample. Furthermore, the specific gravity of the 10:10_5AB and 10:10_10AB samples was higher than that of the 10:6_5AB and 10:6_10AB samples. The epoxy to polyamide ratio of 10:10 had a flexible behavior with large crosslinking in a three-dimensional network in the epoxy resin system, thereby, enabling the droplet to entrap gases and expand [32]. Meanwhile, the PEMBs at the epoxy to polyamide ratio of 10:6 had open pores on their surface (Figure 3), contributing to a decrease in the density of the PEMBs.

### 3.5. Thermal-Gravimetric Analysis (TGA)

**Figure 6:**
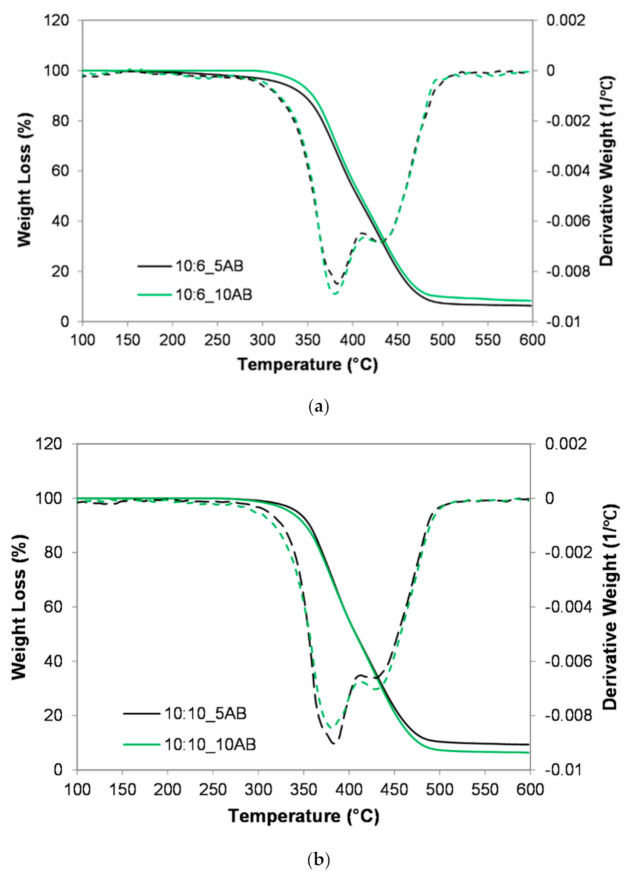
TGA thermograph of PEMBs at different ammonium bicarbonate content and epoxy polyamide ratios: (**a**) epoxy polyamide ratio of 10:6 and (**b**) epoxy polyamide ratio of 10:10.

Figure 6 presents the TGA and DTG analysis of the PEMBs for different ammonium bicarbonate contents and epoxy to polyamide ratios. It was found that the PEMBs exhibited two different slopes related to two different decomposition stages. The first decomposition stage took place at a temperature of around 250–400 °C, which represented the dehydration of the hydroxyl (OH) and carbonyl (C=O) groups of the epoxy structure. Meanwhile, the second decomposition stage at around 400–500 °C represented the breakdown of the carbon backbone of the epoxy structure [33]. The 10:6_10AB and 10:10_5AB samples exhibited a higher thermal stability as compared to the other samples since they had smaller closed-pore micro-beads, which underwent a late onset of decomposition at 297 °C and 272 °C, respectively. However, the 10:6_5AB sample had an earlier onset at 222 °C and a faster decomposition rate. This sample had larger open pore micro-beads with a higher surface area and better heat diffusion [34]. Hence, it exhibited a lower thermal stability. For the effect of the epoxy to polyamide ratio, the 10:6 ratio containing 5 phr of ammonium bicarbonate (10:6_5AB) showed a lower thermal stability. However, it was reported that, with the use of 10 phr ammonium bicarbonate, the sample with an epoxy to polyamide ratio of 10:6 (10:6_10AB) exhibited a higher thermal stability. It was deemed that the 10:6_10AB sample had solid micro-beads, which burned at a slower rate than the porous micro-beads.

## 4. Conclusions

Porous epoxy micro-beads were successfully produced by a single droplet in heated corn oil. The time taken to complete the decomposition reaction was faster with 10 phr of ammonium bicarbonate and a higher total volume of gases was generated. As a result, porous epoxy micro-beads with a smaller particle size, and lower specific gravity and thermal stability were produced. The epoxy to polyamide ratio of 10:6 produced many smaller-sized particles between 201 and 400 μm for both samples of 10:6_5AB and 10:6_10AB. This smaller particle size with a small, open pore decreased the specific gravity and increased the thermal stability of the micro-beads.

## Data Availability

Data is contained within this article.
